# First person – Celia Cordero-Sanchez

**DOI:** 10.1242/dmm.043349

**Published:** 2019-12-03

## Abstract

First Person is a series of interviews with the first authors of a selection of papers published in Disease Models & Mechanisms, helping early-career researchers promote themselves alongside their papers. Celia Cordero-Sanchez is joint first author on ‘A luminal EF-hand mutation in STIM1 in mice causes the clinical hallmarks of tubular aggregate myopathy’, published in DMM. Celia is a PhD student in the lab of Armando Genazzani at the University of Piemonte Orientale, investigating a mouse model of tubular aggregate myopathy.


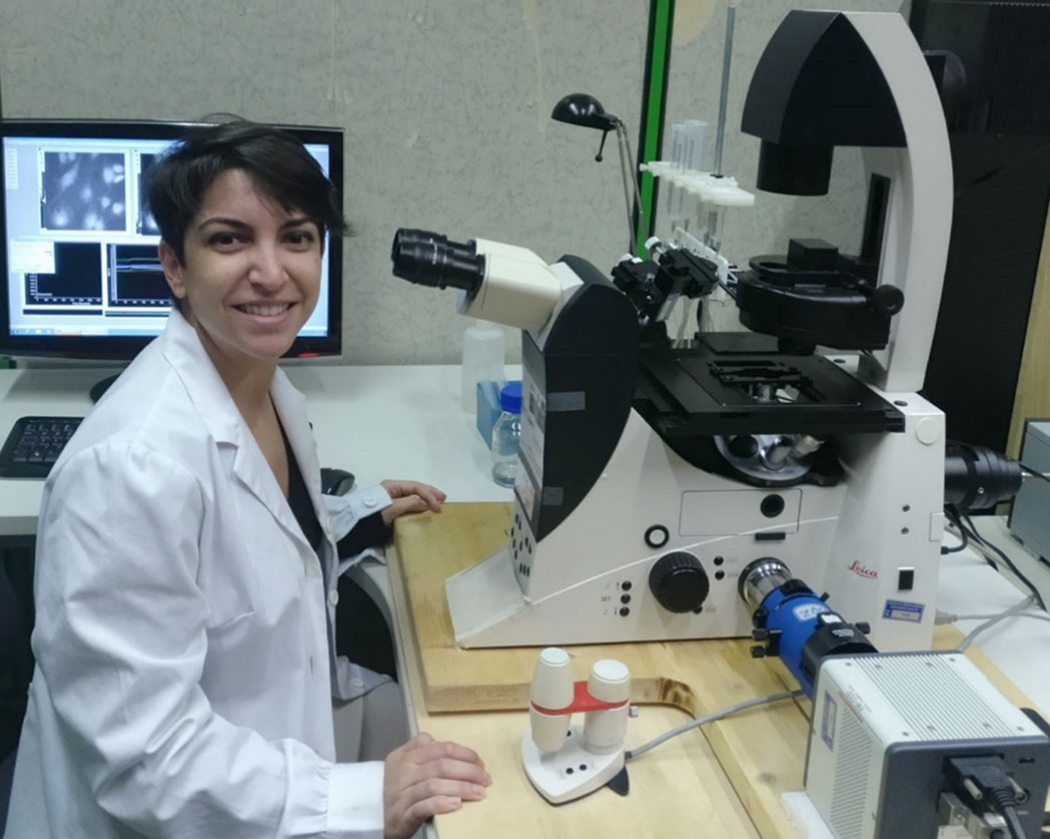


**Celia Cordero-Sanchez**

**How would you explain the main findings of your paper to non-scientific family and friends?**

Tubular aggregate myopathy (TAM) is a rare genetic disease, caused by point mutations in one of the two key proteins (ORAI1 and STIM1) involved in store-operated calcium entry, an intracellular mechanism that regulates the calcium level inside cells. These mutations provoke increased activity of the mechanism, enhancing the level of intracellular calcium.

In our work, we have demonstrated that a mutation of STIM1 also evokes TAM in mice. In this way, we created and validated a mouse model that allows us to study and understand the underlying mechanisms of TAM. Using the model, we evaluated the characteristics of muscles and platelets, which are mainly affected by this disease.

**What are the potential implications of these results for your field of research?**

The creation and validation of animal models are crucial for the development of new therapies for patients. In the case of TAM, there is an imminent unmet medical need for patients that currently receive only symptomatic therapy. The rareness of the illness and the variety of mutations in the two causative proteins make the development of a therapy problematic. In the future, we aim to initiate treating these experimental mice with putative compounds, performing all the pertinent long-term studies to guarantee safety and efficacy. Once at least one of our compounds overcomes all checks, the goal will be to continue with a clinical trial, providing treatment to patients.

**What are the main advantages and drawbacks of the model system you have used as it relates to the disease you are investigating?**

From my personal perspective, the principal advantage of our mouse model is its survival, which allows treatment in the long term, simulating patients' conditions, without excessive suffering of the animals. The principal drawback of this mouse model could be the absence of full aggregates in the muscle structure, a distinctive trait of TAM, although we have observed pseudo-aggregates and structural disorder.

“[…] the principal advantage of our mouse model is its survival, which allows treatment in the long term, simulating patients’ conditions, without excessive suffering of the animals.”

**What has surprised you the most while conducting your research?**

It was a surprise to obtain the pseudo-aggregates in the muscles. These have not been described before and are an interesting aspect of the cellular mechanisms of TAM that should be studied, in order to better understand the illness.

**Describe what you think is the most significant challenge impacting your research at this time and how will this be addressed over the next 10 years?**

The most significant challenge is performing the experiments of the putative treatment in mice. It will be a huge undertaking that will require major synchronization and collaboration. I hope that in 10 years we will be able to conduct the clinical trials.

**What changes do you think could improve the professional lives of early-career scientists?**

As far as I am concerned, it is crucial to have scientific and personal stimulus. The first years provide clues for our learning and the professional route we will take. Collaborations, moving abroad and learning new techniques are fundamental for enriching our growth as researchers, and for that, I would like to express my gratitude to our collaborators that made this work possible. I would particularly like to thank Armando Genazzani, for the opportunity to develop myself as an early-career researcher with my first ‘first-author’ paper, and also Beatrice Riva, for backing me.

**Figure:**
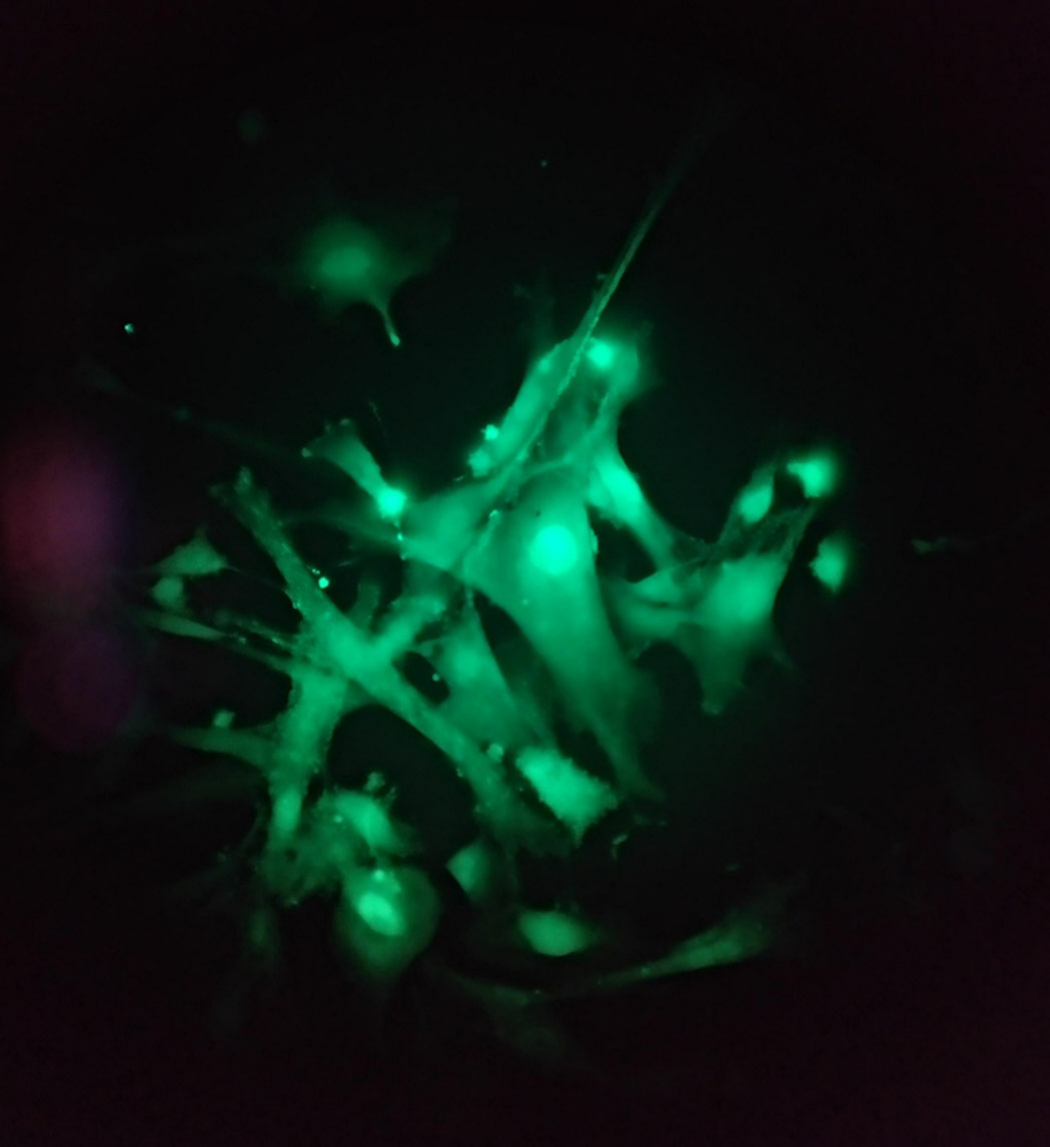
**Mouse muscle cells charged with a calcium fluorescent probe on the microscope.**

**What's next for you?**

I am initiating my second year of my PhD, which will include further characterization of the mouse model and the *in vivo* characterization of the hit compounds for treatments.
